# Identification and Characterization of a Novel Linear B-Cell Epitope Within the ASFV pB602L Protein for Serological Diagnosis

**DOI:** 10.3390/microorganisms14071391

**Published:** 2026-06-23

**Authors:** Biru Chen, Jingming Zhou, Hongliang Liu, Xiao Liu, Haili Wang, Linyi Bai, Jiaojiao Wei, Yaxin Guo, Yidi Lu, Aiping Wang

**Affiliations:** 1 Longhu Laboratory of Advanced Immunology, Zhengzhou 450046, China; 2 School of Life Sciences, Zhengzhou University, Zhengzhou 450001, China; 3 Henan Provincial Key Laboratory of Immunological Biology, Zhengzhou 450001, China

**Keywords:** African swine fever virus pB602L, novel linear B-cell epitopes, recombinant expression, ELISA detection

## Abstract

African swine fever in both domestic and wild pig populations is caused by the extremely infectious African swine fever virus (ASFV). It seriously endangers biodiversity and results in large financial losses for the worldwide pork sector. The major capsid protein p72 is molecularly chaperoned by the ASFV pB602L protein, which is essential to viral assembly. Furthermore, as a nonstructural protein expressed at late stages of infection, pB602L induces a distinct antibody response that may complement existing serological assays based on structural proteins. Given its strong immunogenicity, pB602L represents a promising antigen for developing supplementary diagnostic tools for African swine fever (ASF). In this study, we successfully generated and separated the ASFV pB602L protein, and we verified its responsiveness using serum from pigs infected with ASFV. Additionally, we produced four monoclonal antibody-specific hybridoma cell lines that targeted the pB602L protein exclusively. These cell lines demonstrated high immunoreactivity and responsiveness toward ASFV pB602L. These results highlight the potential enhancement of diagnostic skills. We have detected two previously unknown linear B-cell epitopes (^138^TIDSFL^143^ and ^164^TNVDTC^169^) using overlapping peptide and truncated protein fragment analysis. Due to their high degree of conservation across various ASFV strains, these epitopes offer trustworthy candidates for the creation of particular diagnostic instruments. This study expands the known ASFV antigenic repertoire by systematically mapping immunodominant epitopes of pB602L. The identified epitopes provide potential molecular targets for the rational design of multi-epitope subunit vaccines.

## 1. Introduction

The African swine fever virus (ASFV) is the underlying cause of African swine fever (ASF) [1], a highly fatal viral disease affecting pigs. It began as a widespread illness in Africa and has now spread throughout Eurasia since it was first discovered in Kenya in 1921 [2]. In recent years, particularly since it was introduced to China in 2018, it has caused devastating economic losses to the global swine industry. ASFV has a large and complex genomic structure and remarkable environmental resistance, making it the only known DNA virus that requires biological transmission by arthropod vectors [3,4,5]. It spreads through multiple transmission routes and effectively evades host immune clearance. The ongoing devastation caused by ASF has resulted in unprecedented economic losses for the global pork industry. Its pathogen, ASFV, possesses a complex structure and diverse immune evasion mechanisms, resulting in the absence of any commercially available safe and effective vaccine to date [6,7]. Against this grim backdrop, vaccine development has shifted focus from traditional whole virus approaches to more targeted and safer subunit vaccines. The core of subunit vaccine design lies in the precise characterization of key viral antigen proteins and their protective epitopes [8,9,10,11].

A crucial biochemical chaperone and exceptionally conserved late-expression protein, pB602L controls the precise construction and completion of the ASFV capsid [12,13]. Its crucial function throughout the trimerization of the main capsid protein p72, a crucial stage in viral morphological development, is confirmed by experimental data. Inactivation or loss of function of pB602L impedes the folding and polymerization of p72, thereby inhibiting the formation of mature, infectivity-competent viral particles [14,15]. In contrast to early-expressed membrane-associated proteins such as p54 and p30, which primarily function in viral attachment and internalization, pB602L acts at a later stage of morphogenesis. Furthermore, pB602L is not a direct target for neutralization but it elicits a strong response from CD8^+^ T cells [16,17,18]. Animal studies have shown that subunit or DNA vaccines targeting pB602L can provide partial to complete protection against lethal ASFV challenge, underscoring its importance in cellular immunity [7,19]. Therefore, identifying the T-cell and B-cell epitopes of pB602L is critical for clarifying its immune protective mechanisms and guiding the design of next-generation multi-epitope vaccines [12,20,21].

As is well acknowledged, commercial ASF serological diagnostic kits mainly adopt p30, p54, p72 and CD2V antigens and have been widely applied in clinical detection. Meanwhile, although pB602L has traditionally been used as a target for PCR-based nucleic acid detection, multiple recent studies have fully verified that recombinant pB602L can also serve as an effective antigen for ASFV serological antibody detection [13,22,23]. These findings confirm the great potential of pB602L in serological diagnosis, extending its utility beyond nucleic acid detection. Nevertheless, most existing relevant studies have only utilized the full-length pB602L protein to establish ELISA methods, while systematic screening and identification of its dominant B-cell epitopes remain insufficient. As a late-expressed, highly conserved nonstructural protein of ASFV, pB602L can induce persistent antibody responses in infected pigs. Identification of its core immunodominant epitopes could further optimize pB602L-based serological detection reagents, improve diagnostic performance, and simultaneously provide novel epitope candidates for subsequent ASF vaccine development.

This study aims to accurately map the immunodominant antigen spectrum of pB602L by integrating bioinformatics predictions, screening using an overlapping peptide library, and in vitro cellular functional characterization. The two novel epitopes were further characterized using specific monoclonal antibodies (mAbs). Overall, this study expands the antigenic repertoire of ASFV, comprehensively elucidates the immunological features of pB602L, and provides evidence-based support for developing epitope-based multiplex diagnostic platforms.

## 2. Materials and Methods

### 2.1. Materials

The HEK293T (CCTCC No. GDC0187) and SP2/0 (CCTCC No. GDC0019) cell lines used in this study were purchased from purchased from China Center for Type Culture Collection (CCTCC, Wuhan, China) and routinely maintained in our laboratory. Serum samples from pigs that tested positive for ASFV are stored in this laboratory alongside other pig serum samples. HEK293T cells were cultured in DMEM medium (Gibco, Grand Island, NY, USA), supplemented with 10% fetal bovine serum (FBS) (Gibco, Grand Island, NY, USA) and 1% penicillin–streptomycin (Gibco, Grand Island, NY, USA). SP2/0 cells were cultured in RPMI-1640 medium (Gibco, Grand Island, NY, USA) supplemented with 10% FBS and 1% penicillin–streptomycin. Both cell lines were cultured at 37 °C in a 5% CO_2_ incubator. Specific-pathogen-free (SPF) BALB/c mice (6~8 weeks old) were supplied by the Zhengzhou University Laboratory Animal Center (Zhengzhou, China). Molecular cloning vectors, including pET28a(+), pET32a(+), pCDNA 3.1, and PEGFP-C1 were purchased from Tsingke Biotechnology Co., Ltd. (Beijing, China) and maintained in the laboratory. *E. coli* strains DH5α and BL21(DE3) were purchased from WEIDI Biotech (Shanghai, China).

### 2.2. Bioinformatical Analysis and Construction of pB602L Protein Expression Plasmid

The *pB602L* gene (GenBank accession No. PV592808.1) was codon-optimized for expression in *E. coli* and synthesized by Sangon Biotech Co., Ltd. (Shanghai, China). The High Fidelity HS DNA Polymerase Kit (Takara Bio Inc., Dalian, China) was used for standard PCR amplification. The PCR products were separated by agarose gel electrophoresis, and the target DNA fragments were extracted and purified using a gel extraction kit (TianGen, Beijing, China). The *pB602L* gene fragment was ligated into the *BamH* I/*Hind* III double-digested pET-28a (+) vector, and the ligated construct was transformed into competent *E. coli* DH5α cells. Table 1 lists all primers used in this study. Positive clones were confirmed by PCR and validated by DNA sequencing. For protein expression, the sequence-verified recombinant plasmid was transformed into *E. coli* BL21(DE3) cells.

The online tool TMHMM 2.0 (https://services.healthtech.dtu.dk/services/TMHMM-2.0/, accessed on 20 June 2026) was used to predict the transmembrane domains of pB602L. B-cell epitopes of pB602L were predicted using the IEDB database (http://tools.iedb.org/bcell/, accessed on 20 June 2026). MEGA (version 12) alignment was used to examine the conservation of amino acid sequences. AlphaFold3 (https://alphafoldserver.com/, accessed on 20 June 2026) was used to predict the three-dimensional structure of pB602L. PyMOL software (version 3.1.0; Schrödinger, Inc., New York, NY, USA) was used to visualize the predicted structure.

### 2.3. Expression and Purification of Recombinant pB602L Protein

Positive colonies of recombinant *E. coli* BL21(DE3) harboring the pET28a-pB602L plasmid were inoculated into LB liquid medium and cultured at 37 °C until the OD_600_ reached 0.6~0.8. Isopropyl-β-D-1-thiogalactopyranoside (IPTG) was then added to a final concentration of 0.5 mM to induce pB602L protein expression under standard conditions. To achieve maximum soluble expression of the recombinant pB602L protein, a series of expression conditions were subsequently optimized using a factorial design (Table 2). After being extracted, the cells were reintroduced in a conventional lysis buffer (50 mmol/L Tris-HCl, 150 mmol/L NaCl, pH 8.0) and sonicated vigorously for ten to twenty minutes on ice. After that, the sample was centrifuged once more for 25 min at a speed of 10,000 rpm in order to separate inclusion bodies found in the pellet from soluble proteins that exist in the supernatant. Purify the His-tagged pB602L protein from the supernatant by eluting it onto a High Affinity Ni-Charged Resin FF column (GenScript, Nanjing, China) with Tris-HCl buffer. The target protein was gradient eluted using varying imidazole concentrations (10 mM to 500 mM), with eluates collected and analyzed by SDS-PAGE. Eluted fractions were dialyzed into PBS using a 3 kDa MWCO dialysis membrane, followed by concentration using a 30 kDa MWCO centrifugal filtration unit (Merck Millipore, Burlington, MA, USA). Protein concentration was quantified using the BCA Protein Assay Kit (Thermo Fisher Scientific, Waltham, MA, USA). Samples were aliquoted into sterile microcentrifuge tubes and stored at −80 °C for subsequent experiments.

### 2.4. Preparing pB602L Specific Monoclonal Antibodies

**Figure 1:**
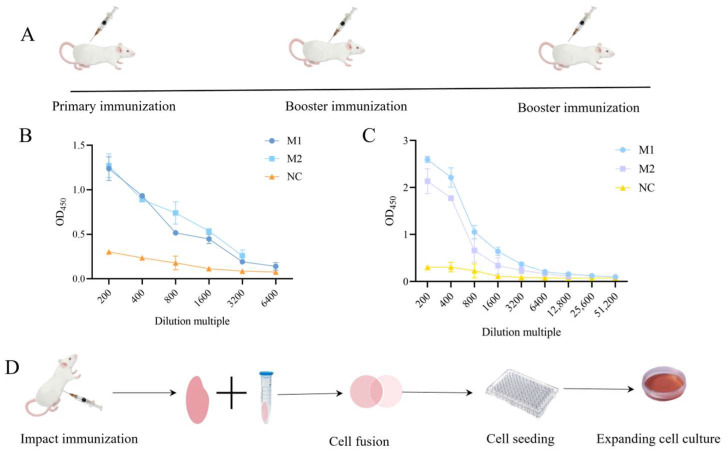
Production of monoclonal antibodies. (**A**) Immunization protocol in BALB/c mice; (**B**,**C**) serum antibody titers measured by indirect ELISA after the first (**B**) and second (**C**) booster immunizations. Data are presented as mean OD_450_ ± SD (*n* = 3); (**D**) screening of hybridoma supernatants for pB602L-specific monoclonal antibodies.

Monoclonal antibodies specific to ASFV pB602L were generated using standard hybridoma technology as previously described. Six-to-eight-week-old female BALB/c mice were immunized subcutaneously at multiple dorsal sites with 20 μg of purified pB602L protein emulsified in Freund’s complete adjuvant (Sigma-Aldrich, Shanghai, China). Two booster immunizations were administered at two-week intervals, each consisting of 20 μg of the same protein emulsified in Freund’s incomplete adjuvant (immunization schedule shown in Figure 1A). The titers of pB602L-specific antibodies in mouse serum were determined by indirect enzyme-linked immunosorbent assay (ELISA). A solution of 50 μg of recombinant protein dissolved in PBS buffer was administered via intraperitoneal injection to mice at high titers (Figure 1B,C). Three to four days after immunization, spleen cells were collected under sterile conditions for use in cell fusion assays. The procedure involved isolating spleens from immunized mice, gently grinding them through a 200-mesh nylon cell strainer, and finally diluting to a standard concentration. Myeloma cells were mixed with spleen cells at a 1:6 ratio. PEG 1500 was then slowly added dropwise to the cell mixture, which was maintained in a 37 °C water bath. The fusion reaction was terminated after 90 s. Cells were diluted in medium and seeded into 96-well plates (Figure 1D). Ten days post-fusion, hybridoma cells were screened using indirect ELISA. Positive hybridoma cells underwent subcloning via serial dilutions in 96-well plates. For large-scale monoclonal antibody production, ascites was induced using liquid paraffin and purified together with monoclonal hybridoma cells.

### 2.5. Cell Transfection and Immunofluorescence Detection

PEI transfection reagent was used to transfect recombinant plasmid pcDNA3.1-pB602L into HEK293T cells that were plated in 6-well plates. For 48 h, cells had been incubated at 37 °C with 5% CO_2_. Following the removal of the medium, cells were fixed with a concentration of and gently rinsed once with cooled phosphate-buffered saline (PBS, pH 7.4). Formaldehyde was applied to the cells for 20 min at room temperature. The cells were then permeabilized with 0.1% Triton X-100 for 20 min, rinsed three times with PBS for five minutes each, and then washed once more with PBS. Cells were treated with PBS containing 5% skim milk at 37 °C for one hour in order to prevent non-specific binding. Next, incubate with anti-pB602L monoclonal antibody or SP2/0 cell supernatant (as negative control) at 37 °C for 1 h. After washing, add FITC-labeled goat anti-mouse IgG antibody (Beijing Bioscience Co., Ltd., Beijing, China).and incubate for another hour. The cell nuclei were incubated with DAPI for 10 min. Fluorescence images were recorded using a Nikon Eclipse Ti2 inverted fluorescence microscope (Tokyo, Japan) and analyzed using NIS-Elements software (version 5.21.00).

### 2.6. Stepwise Truncation and Epitope Mapping of pB602L

Six overlapping peptide segments (designated pB602L-J1 to pB602L-J6) covering the entire amino acid sequence of pB602L were designed and amplified by PCR to precisely map its B-cell epitopes. The PCR products were digested with *EcoR* I and *Xho* I and subsequently cloned into the pET32a (+) prokaryotic expression vector to generate plasmids expressing truncated pB602L proteins. The verified recombinant plasmids were transformed into competent *E. coli* BL21(DE3) cells, and recombinant pB602L protein expression was induced by IPTG. Following induction, the bacterial cells were lysed using sonication, and the supernatant containing soluble recombinant proteins was collected by centrifugation for use in dot-blot analysis and the indirect ELISA. Based on the binding reactivity of the peptide fragments (pB602L-J2, pB602L-J3, and pB602L-J6) with the cell culture supernatant, a second round of truncation was performed. The resulting truncated fragments were subcloned into the pET-32a (+) vector, and their immunoreactivity with the cell culture supernatant was confirmed by indirect ELISA and dot blot analysis. The J2, J3, and J6 derivatives were subjected to further truncation, and their antigenicity was validated using previously characterized monoclonal antibodies (mAbs). The newly identified positive truncated fragments, J10 and J16, were subjected to additional rounds of truncation, and the resulting fragments were cloned into the pEGFP-C1 vector. Immunofluorescence staining was then performed to assess the binding reactivity of these target peptide fragments with the cell culture supernatant.

### 2.7. Dot Blot Assay

Nitrocellulose membranes were cut into adequately sized strips and immersed in PBS buffer for ten minutes in order to detect areas or peptides identified by the pB602L specific monoclonal antibody. Cell lysate from empty vector transfected cells was used as the negative control, and 2 μL of protein sample was spotted onto each well after air-drying at room temperature. The 5% skim milk was used to block the dried spotted membrane for two hours at 37 °C. It was rinsed three times with 1×PBS buffer with 0.05% Tween-20 following the monoclonal antibody incubation. After that, sheep anti-mouse IgG antibody (Beijing Solabio Technology Co., Ltd., Beijing, China) was added, and the mixture was incubated for one hour at 37 °C. Signals were identified using ECL and captured using Tanon imaging after three washes.

### 2.8. Bioinformatics Analysis

For the identified antigenic epitopes, we performed multiple sequence alignment and conservation analysis using the NCBI tool (National Institutes of Health, https://www.ncbi.nlm.nih.gov/, accessed on 20 June 2026). Representative ASFV strains were selected from the NCBI database, and sequence alignment was conducted using the MAGE software (version 12). To characterize the structural features of the epitopes, we employed AlphaFold 3 (https://alphafold3.org/) to predict the three-dimensional structure of the pB602L protein. Spatial localization of the epitopes was visualized using PyMOL software (version 3.1.0; Schrödinger, Inc., New York, NY, USA).

### 2.9. Statistical Analysis

All the data in quantitative graphics of the ELISA-generated line, heat map and histogram were constructed by GraphPad Prism 9.0 (GraphPad Software, San Diego, CA, USA) within three experimental replicates and shown as means ± SD. For single-factor multi-group ELISA data, parametric one-way ANOVA followed by Tukey’s multiple comparisons test was adopted. Unpaired two-tailed Student’s *t*-test was used for comparisons between two groups. *p* values as follows: * *p* < 0.05; ** *p* < 0.01; *** *p* < 0.001.

## 3. Results

### 3.1. pB602L Protein Preparation

**Figure 2:**
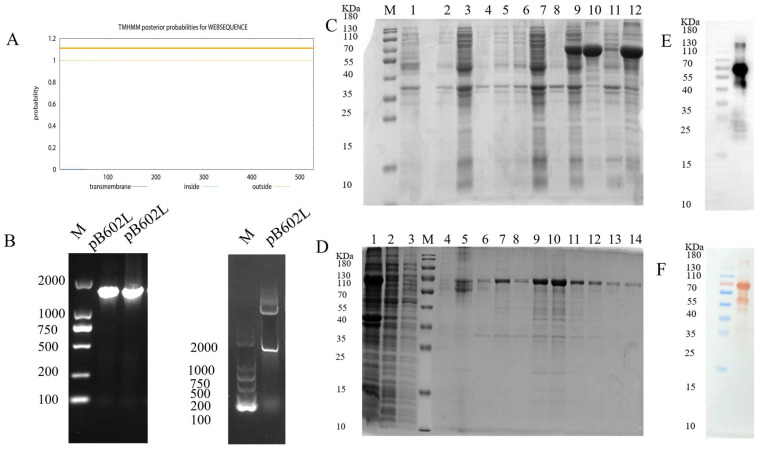
Expression, purification, and characterization of the recombinant pB602L protein. (**A**) Prediction of the transmembrane domains of pB602L, (the bold orange line indicates the probability of being outside the membrane); (**B**) PCR amplification of the pB602L gene and validation by double restriction enzyme digestion of recombinant plasmids. (**C**) pB602L concentrations in the supernatant of lysed *E. coli* under different orthogonal experimental conditions; lane M contains protein markers, lanes 1~12 show the supernatant under different temperatures and IPTG concentrations. (**D**) Purity of the recombinant pB602L protein was determined using a Ni-NTA affinity chromatography method; (**E**) Western blot analysis of recombinant pB602L probed with anti-His tag antibody. The ~70 kDa band is the full-length protein, the 110~130 kDa band is a partially degraded homodimer, additional bands (40~55 kDa) are proteolytic degradation fragments. All bands retain the N-terminal His tag. (**F**) Validation of the PB602L protein using ASFV-positive serum via Western blot, detected using AEC substrate (red-brown precipitate).

Based on the sequence obtained from the NCBI database, we successfully synthesized the full-length pB602L gene of ASFV. As shown in Figure 2A, the tool predicts that this protein lacks transmembrane properties. The target gene was amplified by PCR and cloned into the pET28a (+) prokaryotic expression vector to construct the recombinant plasmid pET28a-pB602L. The recombinant expression vector was verified by double-enzyme restriction digestion (Figure 2B). The verified positive plasmid was transformed into *E. coli* BL21(DE3) competent cells; IPTG was used to induce the expression of the pB602L protein (Figure 2C). We identified the conditions under which albumin expression was highest and induced its expression at high levels. Then, the recombinant protein was subsequently purified via nickel–NTA affinity chromatography. Sodium dodecyl sulfate polyacrylamide gel electrophoresis (SDS-PAGE) analysis with Coomassie Brilliant Blue staining revealed purified pB602L protein with >90% purity and a molecular weight of 68 kDa, consistent with the theoretical value (Figure 2D). Western blot analysis confirmed specific recognition by the anti-His tag antibody and immunoreactivity with ASFV-positive porcine serum (Figure 2E,F). The multiple bands in Figure 2E correspond to full-length pB602L (68 kDa), a partially degraded homodimer (110~130 kDa), and proteolytic fragments (40~55 kDa), all retaining the N-terminal His tag.

### 3.2. Preparation and Characterization of pB602L-Specific Monoclonal Antibodies

**Figure 3:**
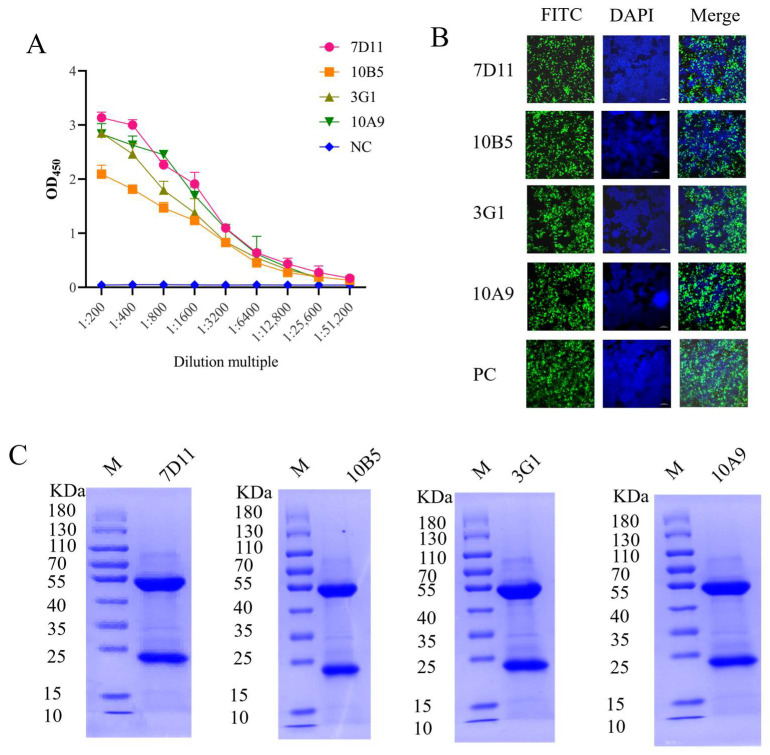
Identification and purification of pB602L-specific monoclonal antibodies. (**A**) Antibody titer in hybridoma cell culture medium; the optical density (OD_450_) values represent the degree of antibody binding. Error bars represent mean ± SD (*n* = 3). (**B**) Determination of the reactivity of this monoclonal antibody with pB602L expressed in HEK293T using immunofluorescence assay (IFA (green: pB602L-FITC; blue: DAPI-stained nucleus)). (**C**) Identified purified monoclonal antibodies using SDS-PAGE.

Following the immunization protocol described in Section 2.4, the antibody titers in the serum of both BALB/c mice exceeded 1:1.28 × 10^4^. Through subcloning, four hybridoma cell lines (7D11, 10B5, 3G1, 10A9) secreting pB602L-specific antibodies were successfully isolated. Antibody titers in cell culture ranged from 1:6.4 × 10^3^ to 1:2.56 × 10^4^ (Figure 3A). HEK293T cells transfected with pcDNA3.1-pB602L-His were fixed 48 h after expression and incubated with the monoclonal antibody for immunofluorescence analysis (Figure 3B). The resulting antibody was subsequently purified using protein A. After purification, the antibody was verified by SDS-PAGE (Figure 3C), revealing characteristic bands at 55 kDa and 25 kDa.

### 3.3. Study on the Localization of B-Cell Epitopes Recognized by Anti-pB602L Protein Monoclonal Antibodies

**Figure 4:**
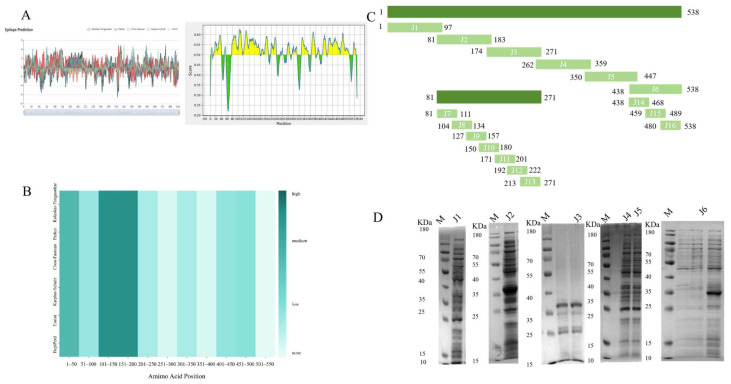
B-cell epitope prediction based on bioinformatics algorithms and a schematic diagram of the truncated pB602L protein. (**A**) B-cell epitope prediction based on multiple bioinformatics algorithms, (regions shaded in yellow are predicted potential B-cell epitopes, while regions shaded in green are non-epitope regions); (**B**) heatmap showing the dominant epitope regions of the pB602L protein; (**C**) schematic diagram of the truncated pB602L protein; (**D**) SDS-PAGE of the truncated pB602L protein expressed in *E. coli*.

First, we predicted potential B-cell epitopes of the pB602L protein using five online algorithms: the Kolaskar–Tongaonkar antigenicity index, Parker hydrophilicity, Chou–Fasman β-turns, Karplus–Schulz flexibility, and Emini surface accessibility. Overlapping high-scoring regions predicted by multiple algorithms were mainly distributed at 30~50 aa, 80~140 aa, 200~240 aa, 310~350 aa, 360 aa~420 aa and 440~500 aa, indicating that these segments possess high antigenicity, hydrophilicity, surface accessibility, and structural flexibility, and are therefore candidate linear B-cell epitopes (Figure 4A). A heatmap analysis was performed on the epitopes predicted by these five methods to identify regions likely to contain dominant epitopes (Figure 4B). The pB602L protein was truncated using the overlapping extension method to cover each epitope region (Figure 4C). The soluble expression of the truncated proteins (segments J1–J6) in *E. coli* was demonstrated (Figure 4D).

**Figure 5:**
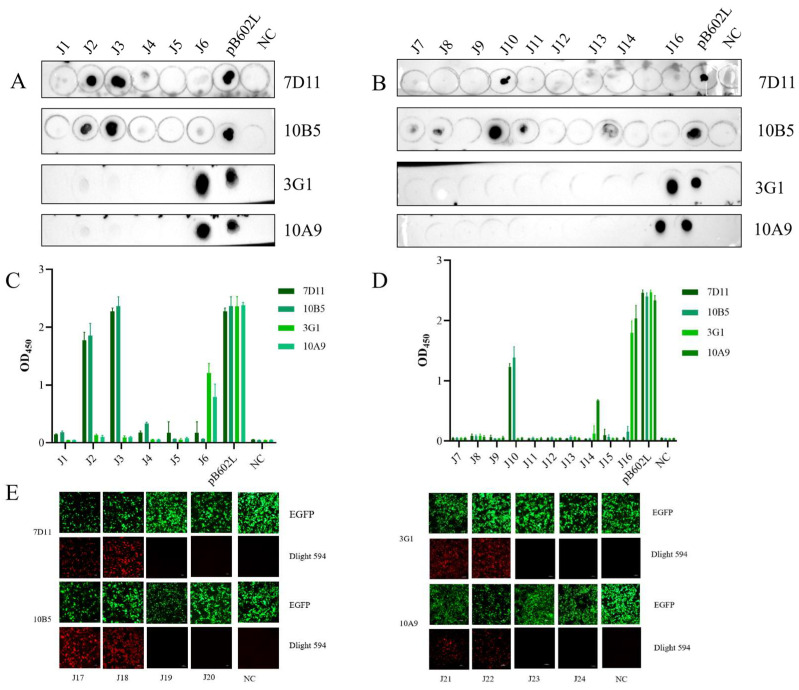
Localization of linear B-cell epitopes on the pB602L protein. (**A**,**B**) Dot-blot analysis of monoclonal antibody reactivity with truncated pB602L proteins J1~J6 (**A**) and J7~J16 (**B**); (**C**,**D**) indirect ELISA of monoclonal antibody reactivity with J1–J6 (**C**) and J7–J16 (**D**). OD_450_ values represent mean ± SD (*n* = 3 biological replicates); (**E**) IFA of the reaction between the monoclonal antibody and the pB602L truncated protein (J17~J24) (green fluorescence represents the truncated pB602L protein, and red fluorescence indicates the fluorescent secondary antibody).

Dot blot analysis of these sub-fragments revealed that monoclonal antibodies 7D11 and 10B5 specifically bound J10, while 3G1 and 10A9 specifically bound J16 (Figure 5A,B); these results were confirmed by ELISA (Figure 5C,D). To precisely define the epitope sequences, we further truncated J10 and J16 into smaller fragments (J17~J24) and performed immunofluorescence analysis in 293T cells expressing J17~J24-EGFP fusion proteins. The results showed clear colocalization of 7D11 and 10B5 with J17 and J18 (Figure 5E), and clear colocalization of 3G1 and 10A9 with J23 and J24. Because J17 and J18 overlap by six amino acids, and J23 and J24 also overlap by six amino acids, the sequences recognized by 7D11 and 10B5 contain a naturally occurring linear B-cell epitope (^138^TIDSFL^143^), whereas the epitope recognized by 3G1 and 10A9 was designated as ^164^TNVDTC^169^.

**Figure 6:**
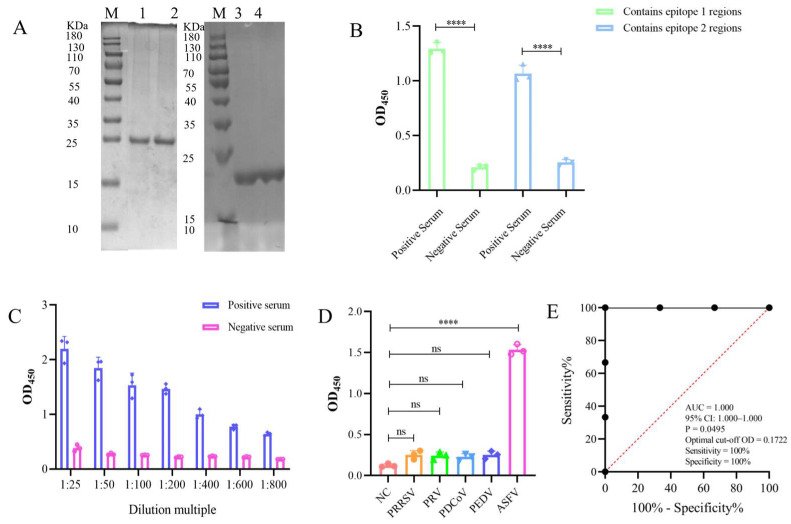
Development of an indirect epitope ELISA for the detection of African swine fever-specific antibodies. (**A**) Purification of the epitope-containing protein (lanes 1 and 2 contain the epitope region ^138^TIDSFL^143^; lanes 3 and 4 contain the epitope region ^164^TNVDTC^169^; M denotes the protein marker). (**B**) Coating with the epitope-containing protein at a concentration of 1 ng/µL. The epitope-based ELISA was performed to test ASFV-positive and negative sera at a serum dilution of 1:400. Data were obtained from at least three biological replicates (mean ± SD). Statistical significance was analyzed by unpaired *t*-test (**** *p* < 0.0001). Error bars represent mean ± SD. (**C**) Serum dilutions tested by the indirect ELISA. The optical density (OD_450_) value reflects the extent of antibody binding. Data are presented as mean ± standard deviation (*n* = 3). (**D**) Specificity of the established indirect epitope ELISA. PRRSV-, PRV-, PDCoV-, PEDV-, and ASFV-positive and negative pig sera. OD_450_ absorbance values were determined by indirect ELISA, with three biological replicates for each group. Error bars represent mean ± SD. One-way ANOVA followed by Tukey’s multiple comparisons test was used for statistical analysis (ns, no significant difference; **** *p* < 0.0001; NC, negative control). (**E**) ROC curve analysis evaluating diagnostic performance of the epitope-based indirect ELISA for ASFV antibody detection in porcine serum. The black solid curve represents the assay’s diagnostic capacity; the red dashed diagonal line indicates random classification (AUC = 0.5). AUC = 1.000 (95% CI: 1.000~1.000, *p* = 0.0495). At the optimal OD cut-off of 0.1722, the ELISA achieved 100% sensitivity (95% CI: 43.85%~100.0%) and 100% specificity (95% CI: 43.85%~100.0%). The wide confidence intervals of diagnostic indices originate from the limited sample size (*n* = 3).

To determine whether these two epitopes could be used for diagnostic purposes, proteins containing the truncated epitopes were expressed and purified (Figure 6A). The truncated epitope-containing proteins were used as coating antigens at a concentration of 1 ng/µL, and African swine fever-positive and -negative sera were diluted at a 1:400 ratio for use in an indirect ELISA to detect serum antibodies. The results showed that both epitope-containing proteins could be used to detect antibodies in ASF-positive sera (Figure 6B). To investigate the feasibility of this method, we continued to explore the optimal dilution ratio for the serum. The best detection performance was observed when ASFV-positive serum was diluted 1:25 (Figure 6C). When an ELISA was established using the epitope-containing proteins to detect antibodies in ASFV-, PRRSV-, PRV-, PDCoV-, and PEDV-positive pig sera, ASFV-positive sera were successfully detected (Figure 6D). However, given the small sample size (*n* = 3 per group), further validation with larger panels is required to confirm specificity.

### 3.4. Visualization and Conservation Analysis of the pB602L Protein Structure

**Figure 7:**
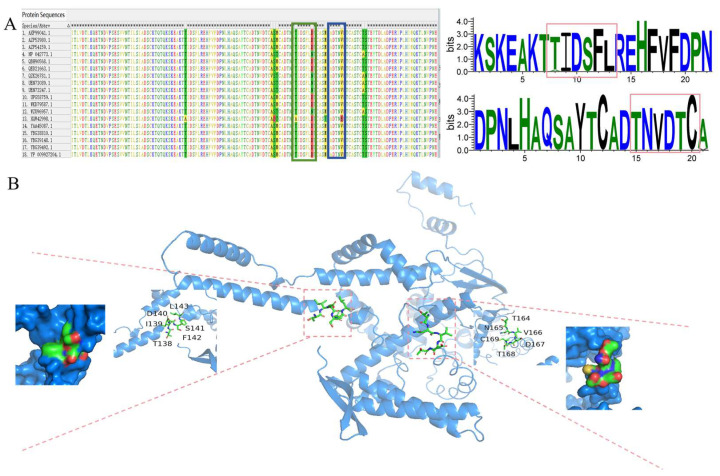
Analysis of the conservation of three linear B-cell epitopes on the pB602L protein and 3D model visualization. (**A**) Analysis of epitope conservation. Red boxes in sequence logos mark conserved epitope regions, which are highlighted by green and blue boxes in multiple sequence alignment. Amino acids are colored by physicochemical properties: red (positive), blue (negative), green (polar uncharged), yellow (hydrophobic), magenta (glycine). Asterisks mark fully conserved sites. The green bar indicates a highly conserved column with one alanine mutation highlighted in pale yellow. The height of each amino acid letter reflects site conservation, with residues colored by physicochemical properties (blue: basic, green: polar neutral, black: hydrophobic, red: acidic residues); (**B**) 3D model of the epitope visualized using PyMOL software. The pB602L protein backbone is rendered as light blue ribbons. Key epitope residues are shown in stick representation with the following color scheme: carbon in green, oxygen in red, nitrogen in blue, and sulfur in yellow. The colored regions indicate the spatial distribution of the candidate linear B-cell epitopes.

To assess the conservation of the newly identified linear B-cell epitope, we retrieved pB602L protein amino acid sequences from the NCBI database and performed conservation analysis using MEGA for multiple sequence alignment. Results revealed that the epitope sequences ^138^TIDSFL^143^ and ^164^TNVDTC^169^ were highly conserved across the vast majority of pB602L strains (Figure 7A). To further characterize its structural features, we predicted the three-dimensional structure of the pB602L protein using the latest AlphaFold 3 algorithm and visualized it with PyMOL. Structural analysis revealed that this epitope is located on the protein surface, exhibiting a typical linear epitope conformation—an α-helix and flexible loop structure (Figure 7B). This unique spatial arrangement may be the key factor underpinning its strong antigenicity and evolutionary conservation.

## 4. Discussion

Previous attempts to manufacture subunit vaccines based on the primary capsid protein p72 have faced significant challenges since p72 by itself is unable to form native peptide capsomers and needs an additional chaperone role for proper folding [24]. As a result, p72-mediated vaccines administered via Modified Vaccinia Ankara (MVA) or Newcastle Disease Virus (NDV) vectors have not been able to prevent deadly ASFV infection in pigs or produce measurable antibody responses [25,26,27]. On the other hand, pB602L, which is the natural chaperone of p72, is a particularly immunogenic antigen that is essential to the assembly of the viral capsid [28,29]. Therefore, identifying and evaluating the specific epitopes of pB602L that contribute to its immunogenicity and chaperone function may facilitate the design of more effective subunit vaccines against ASFV.

Epitope-centric design has emerged as a promising strategy for next-generation ASF vaccines, with mounting preclinical evidence supporting its translational value. Song et al. constructed a self-assembled nanoparticle vaccine (NanoFVax) carrying dominant B- and T-cell epitopes derived from p72, CD2v, pB602L and p30, which elicited sustained high-level antibody responses over 231 days in animal models [21]. Analogously, Duan et al. generated a dendritic cell-targeted biomimetic nano vaccine incorporating B- and T-cell epitopes of p30, p54, p72, pB602L and CD2v, which triggered potent humoral and cellular immunity in mice [30]. Additionally, an mRNA cocktail vaccine encoding pB602L, CD2v, EP153R, p30, p54, and p72 was demonstrated to induce robust multivalent immune responses in both mice and swine [31]. Collectively, these studies verify that precisely mapped B- and T-cell epitopes serve as modular, rationally designed building blocks for multi-epitope ASF vaccines. Notably, most existing multi-epitope platforms employ full-length pB602L rather than the minimal immunodominant epitopes identified through in vitro functional screening. The linear B-cell epitopes characterized in our work can streamline vaccine construction and reduce the antigenic burden on delivery vectors.

In this study, we successfully achieved soluble expression of the recombinant pB602L protein using the Escherichia coli system. This expression platform offers advantages such as high yield, low cost, a clear genetic background, and simple purification, although it lacks post-translational modifications and carries a risk of endotoxin contamination. Notably, previous studies have often expressed ASFV pB602L or its homologs as inclusion bodies. Here, we obtained soluble pB602L to a large extent while retaining sufficient immunological activity, as demonstrated by strong reactivity with four monoclonal antibodies, indirect ELISA and dot blot assays. This makes the recombinant protein highly suitable as a coating antigen for ASFV serological assays and as an immunogen for antibody generation.

To map the linear B-cell epitopes of pB602L, we employed a combination of bioinformatic prediction and overlapping polypeptide design—an approach that is methodologically suitable and highly efficient for initial broad-scale epitope mapping. Five complementary algorithms were used to predict candidate regions, and the predicted high-scoring regions were subsequently validated by systematic truncation and overlapping peptide mapping using four monoclonal antibodies. The consistency between computational predictions and experimental data confirms the reliability of this combined approach. Nevertheless, an inherent limitation of this approach is its inability to capture conformational or discontinuous epitopes.

Using overlapping synthetic peptides covering the entire pB602L sequence, we identified two linear B-cell epitopes at specific amino acid positions: ^138^TIDSFL^143^, and ^164^TNVDTC^169^. Notably, both identified epitopes are predominantly located in surface-exposed regions of the protein, consistent with their high immunogenicity. A previous study by Song et al. identified a conserved B-cell epitope ^474^SKENLTPDE^482^ in the C-terminus of pB602L using a pB602L-specific monoclonal antibody [32]. Our findings complement this existing knowledge by systematically mapping multiple immunodominant regions across the pB602L sequence.

Recently, alternative epitope discovery strategies have been reported for other ASFV antigens. In addition to the epitope identified in the present study, Ma et al., characterized a linear epitope on the ASFV DP238L protein using phage random peptide library display technology, providing an alternative antigen candidate for ASFV serological detection [33]. Different from the phage display strategy applied in their work, we utilized a series of overlapping truncated peptides to narrow down the minimal linear epitope of pB602L, an ASFV protein with distinct functional properties from DP238L. In terms of diagnostic performance, the DP238L epitope-based ELISA achieved acceptable diagnostic efficiency but showed mild cross-reactivity against partial heterologous swine virus sera. In contrast, our pB602L epitope exhibited undetectable background signals when incubated with PRRSV-, PRV-, PDCoV- and PEDV-positive sera. ROC curve analysis in this preliminary cohort (*n* = 3 per group) suggested excellent discriminative potential, with an AUC of 1.000 and observed sensitivity/specificity of 100% under these limited sample conditions. Notably, consistent with the limitation acknowledged by Ma et al. [33], our preliminary diagnostic evaluation was restricted by a very small serum sample cohort (*n* = 3 per group), which leads to wide confidence intervals and reduced statistical power. Therefore, these performance metrics should be interpreted as exploratory rather than definitive. Further validation with a larger panel of field clinical sera is required to confirm the universal diagnostic value of this pB602L epitope in future research.

The identification of these surface-localized epitopes has important implications for vaccine design. Recent studies have demonstrated that antibodies against p30 can mediate antibody-dependent cellular cytotoxicity (ADCC) [20], underscoring the functional relevance of antibody responses targeting ASFV structural proteins. Given that pB602L is a late nonstructural protein involved in viral assembly, epitopes derived from pB602L may offer distinct advantages when combined with epitopes from structural proteins such as p30, p54, or p72 [10,34,35,36]. We propose that rationally combining the pB602L-derived epitopes identified here with epitopes derived from other ASFV proteins could enable a dual-targeted vaccine strategy that simultaneously disrupts viral assembly and compromises virion structure. Such a strategy could overcome the limitations of current inactivated and attenuated live vaccines, which offer suboptimal protection and carry risks of chronic infection or reversion to virulence. Thus, the epitopes identified in this study not only expand the ASFV antigenic repertoire but also provide defined molecular targets for the rational design of multi-epitope subunit vaccines, complementing ongoing efforts to overcome the limitations observed with p72-based immunogens.

## 5. Conclusions

In this study, we successfully obtained high-yield soluble recombinant ASFV pB602L protein and screened four specific monoclonal antibody strains targeting pB602L. More importantly, we identified two previously unreported linear B-cell epitopes: ^138^TIDSFL^143^, and ^164^TNVDTC^169^. The antigenic epitopes identified in this study can be used in ELISAs to detect ASFV antibodies. These newly discovered epitopes are expected to be used in the development of epitope-based diagnostic platforms and to provide a basis for the rational design of multi-epitope candidate vaccines against ASFV.

## Figures and Tables

**Table 1:** PCR primers for amplifying the deletion mutant pB602L protein.

Primer	Sequence of Nucleotide (5′ → 3′)
pB602L F	CGCGGATCCATGCATCACCACCACCACCAC
pB602L R	CCCAAGCTTTAACAGTTCAGCCTTTGTGTA
J1 F	CGCGGATCCATGCATCACCACCACCACCAC
J1 R	CCCAAGCTTTAACAGGGTCTTGGTAGTGTG
J2 F	CGCGGATCCATGAAGCCAACCCACACTACC
J2 R	CCCAAGCTTTAAATCCACGTTAGTGTCGGC
J3 F	CGCGGATCCATGAGCATGTGCGCCGACACT
J3 R	CCCAAGCTTTAACTTGAAGGCACGTGGGTT
J4 F	CGCGGATCCATGATGCTCGAGAACCCACGT
J4 R	CCCAAGCTTTAACAGCTGGATGGAGCTAAC
J5 F	CGCGGATCCATGTTCTCTGAAGTTAGCTCC
J5 R	CCCAAGCTTTAACAGTTCCTTGAAGCTCAG
J6 F	CGCGGATCCATGAAGGGTCTGCTGAGCTTC
J6 R	CCCAAGCTTTAACAGTTCAGCCTTTGTGTA
J7 F	CGCGGATCCATGAAGCCAACCCACACTACC
J7 R	CCCAAGCTTTAACTCAGAAGGCACGTCGTT
J8 F	CGCGGATCCATGACTAACGACGTGCCTTCT
J8 R	CCCAAGCTTTAAGGCTTCCTTAGACTTCTG
J9 F	CGCGGATCCATGACCCAGAAGTCTAAGGAA
J9 R	CCCAAGCTTTAAAGCTGACTGGGCGTGCAG
J10 F	CGCGGATCCATGAACCTGCACGCCCAGTCA
J10 R	CCCAAGCTTTAAGCAAGTATCCACGTTAGT
J11 F	CGCGGATCCATGGACACTAACGTGGATACT
J11 R	CCCAAGCTTTAAGTCGGTGTATTCGGTTGA
J12 F	CGCGGATCCATGACTTCAACCGAATACACC
J12 R	CCCAAGCTTTAACTGGAGTTCGTTAGGCAC
J13 F	CGCGGATCCATGAACGTGCCTAACGAACTC
J13 R	CCCAAGCTTTAACTTGAAGGCACGTGGGTT
J14 F	CGCGGATCCATGAAGGGTCTGCTGAGCTTC
J14 R	CCCAAGCTTTAATTCACAGTCGTCGATGAA
J15 F	CGCGGATCCATGTTCATCGACGACTGTGAA
J15 R	CCCAAGCTTTAACAGAGCGTTGTCGAGCTG
J16 F	CGCGGATCCATGACTATCCGTGTTGACATG
J16 R	CCCAAGCTTTAACAGTTCAGCCTTTGTGTA

**Table 2:** Orthogonal experiments to optimize expression conditions.

	Temperature for Expression (°C)	Concentration of IPTG (mM)
Lane 1	16	0.2
Lane 2	16	0.3
Lane 3	16	0.4
Lane 4	16	0.5
Lane 5	16	0.6
Lane 6	16	0.8
Lane 7	25	0.2
Lane 8	25	0.3
Lane 9	25	0.4
Lane 10	25	0.5
Lane 11	25	0.6
Lane 12	25	0.8

## Data Availability

The original contributions presented in this study are included in the article. Further inquiries can be directed to the corresponding author.

## Abbreviations

The following abbreviations are used in this manuscript:

ASFV	African swine fever virus
ASF	African swine fever
mAbs	monoclonal antibodies
FBS	fetal bovine serum
IPTG	isopropyl β-D-1-thiogalactoside
ELISA	Enzyme-linked immunosorbent assay
PCR	polymerase chain reaction
PBS	phosphate-buffered saline
ADCC	antibody-dependent cellular cytotoxicity
SDS-PAGE	sodium dodecyl sulfate polyacrylamide gel electrophoresis
IFA	immunofluorescence assay
